# Genome-wide association study suggests common variants within *RP11-634B7.4* gene influencing severe pre-treatment pain in head and neck cancer patients

**DOI:** 10.1038/srep34206

**Published:** 2016-09-27

**Authors:** Cielito C. Reyes-Gibby, Jian Wang, Mary Rose T. Silvas, Robert K. Yu, Ehab Y. Hanna, Sanjay Shete

**Affiliations:** 1 Department of Emergency Medicine, The University of Texas MD Anderson Cancer Center, Houston, Texas 77030, USA; 2 Department of Biostatistics, The University of Texas MD Anderson Cancer Center, Houston, Texas 77030, USA; 3 Department of Head & Neck Surgery, The University of Texas MD Anderson Cancer Center, Houston, Texas 77030, USA; 4 Department of Epidemiology, The University of Texas MD Anderson Cancer Center, Houston, Texas 77030, USA

## Abstract

Pain is often one of the first signs of squamous cell carcinoma of the head and neck (HNSCC). Pain at diagnosis is an important prognostic marker for the development of chronic pain, and importantly, for the overall survival time. To identify variants influencing severe pre-treatment pain in 1,368 patients newly diagnosed with HNSCC, we conducted a genome-wide association study based on 730,525 tagging SNPs. The patients were all previously untreated for cancer. About 15% of the patients had severe pre-treatment pain, defined as pain score ≥7 (0 = “no pain” and 10 = “worst pain”). We identified 3 common genetic variants in high linkage disequilibrium for severe pre-treatment pain, representing one genomic region at 1q44 (rs3862188, *P* = 3.45 × 10^−8^; rs880143, *P* = 3.45 × 10^−8^; and rs7526880, *P* = 4.92 × 10^−8^), which maps to the *RP11-634B7.4* gene, a novel antisense gene to three olfactory receptor genes. Olfactory receptor genes, upstream effectors of the *MAPK* signaling cascade, might be novel target genes for pain in HNSCC patients. Future experimental validation to explore biological mechanisms will be key to defining the role of the intronic variants and non-coding RNA for pain in patients with HNSCC.

Head and neck cancer is the sixth most common malignancy worldwide. Squamous cell cancer of the head and neck (HNSCC), the most common head and neck cancer, includes cancers of the oral cavity (including the gums and tongue), pharynx, and larynx. In the US, more than 61,760 men and women are expected to be diagnosed with HNSCC in 2016^1^. The 5-year survival rates are respectively 66% and 63% for individuals with cancer of the oral cavity and pharynx and for those with cancer of the larynx^1^. For surviving patients, controlling the symptoms associated with HNSCC and with related therapy are important goals.

Pain is often one of the first signs of HNSCC, with up to 50% of newly diagnosed patients presenting with pain^2^. For some 36% of patients, pain persists throughout cancer progression until 6 months after treatment^2^. Pain at diagnosis is an important prognostic marker for the development of chronic pain, and importantly, for the overall survival time. Overall, the 5-year survival rate in a total of 2,340 HNSCC patients has been found to vary by the presence of pre-treatment pain for those with oral (severe pain = 31% versus non-severe = 52%; p < 0.001) and pharyngeal cancer (severe pain = 33% versus non-severe = 53%; p < 0.001). Even after accounting for disease stage and clinical factors, pre-treatment pain persisted as an independent prognostic factor for survival^3^.

Pain is an increasing clinical challenge, especially among cancer patients since traditional therapies are only partially effective. Furthermore, substantial inter-individual variability is observed in pain sensitivity and analgesic response^4^,^5^. Adding to the complexity is that opioids, the drug of choice for cancer pain, can be neurotoxic, with repeated dose escalation leading to increased tolerance. Precision medicine, which aims to consider each patient’s genetic, environmental and lifestyle characteristics when developing and assigning treatment, represents a valuable goal for patients with cancer-related pain^6^. In this study, we conducted a genome-wide association study (GWAS) of pre-treatment pain in HNSCC patients.

## Results

Patients with HNSCC (n = 1,384) and information on pre-treatment pain were ascertained at the MD Anderson Cancer Center Head and Neck Surgery Clinic. All patients were self-reported Caucasians. A majority of the patients were male (78.4%). The patients also reported comorbidities, including hypertension (40.8%), heart disease (17.2%), lung disease (10.7%), diabetes (9.8%), stroke (4%) and liver disease (3.2%). TNM classification (tumor [T], node [N], and metastasis [M]) is the single most important prognostic factor and treatment determinant in HNSCC, which includes information on the primary tumor, lymph node involvement and distant metastasis^3^. Among the study population, about half of the patients had a relatively large tumor size and/or extension of the primary tumor (T3 or T4; 46.8%); about one third of the patients had tumor cells absent from regional lymph nodes (31.8%); while only 0.3% of the patients presented with metastasis to distant organs. We used the World Health Organization three-step ladder^7^ to categorize the pain medications reported by patients at presentation to MD Anderson Cancer Center. Specifically, 13.2% of patients used nonopioid medication such as aspirin, acetaminophen, or nonsteroidal anti-inflammatory drugs, 31.7% used weak opioids such as codeine and 34.8% used powerful opioids such as morphine.

From that group of 1,384 patients, we randomly selected individuals and assigned them to the discovery (~70%) and replication (~30%) phases of the GWAS by conditioning on identical percentages of males and females and similar distributions of age at diagnosis in the two phases. We excluded 16 individuals due to missing genotypes. The discovery phase had 958 total individuals, with 148 cases (103 male, 45 female; mean age = 57 years; standard deviation [sd] = 13) and 810 controls (628 male, 182 female; mean age = 58 years; sd = 11). The replication phase had 410 total individuals, with 58 cases (42 male, 16 female; mean age = 57 years; sd = 11) and 352 controls (287 male, 65 female; mean age = 58 years; sd = 11). Based on the identity-by-state distances, no hidden population substructures were detected and all patients were identified to have Western European ancestry. After applying standard quality control processes, we had 714,494 tagging single nucleotide polymorphisms (SNPs) available for a total of 1,368 HNSCC patients.

Based on the joint genotype data set from both the discovery and replication phases, we identified 38 SNPs with *P* values < 0.05 in both phases of the study (see Supplementary Table S1), among which we identified three SNPs, rs3862188, rs880143 and rs7526880, representing one genomic region that satisfied the genome-wide statistical significance threshold (*P *< 5 × 10^−08^; Table 1). For SNPs rs3862188, rs880143 and rs7526880, the joint-analyses-based *P* values were 3.45 × 10^−8^, 3.45 × 10^−8^ and 4.92 × 10^−8^, respectively. The joint-analyses-based odds ratios (ORs) for the association between the three SNPs and pre-treatment pain in HNSCC patients were 1.87 (95% confidence interval [CI]: 1.50–2.33), 1.87 (95% CI: 1.50–2.33), and 1.87 (95% CI: 1.49–2.33), respectively. The three SNPs are in high linkage disequilibrium, *r*^*2*^ = 0.99, leading to very similar association results (see Table 1 for phase-specific results).

To identify genes close to these three SNPs, we investigated a 1M region (i.e., 500 kb on each side) using the NCBI map viewer (annotation release 105)^8^. There are 30 genes that map to this region, among which 27 genes belong to olfactory receptor families. Other genes mapping to this region include *NLRP3, GCSAML* and *TRIM58*.

## Discussion

Pain prior to treatment varies among patients with HNSCC when they present in the clinic. Pain level variability can be due to several factors such as site and stage of disease^3^,^9^. In this study, we investigated common genetic markers associated with pain prior to any cancer treatment through a GWAS. We identified chromosome 1:44q as a susceptibility locus, with three SNPs, rs3862188, rs880143 and rs7526880, localizing in the *RP11-634B7.4* gene. This is a novel finding for pre-treatment cancer pain, and to our knowledge, this is the first study to suggest a clinical significance for these SNPs.

The *RP11-634B7.4* gene has two transcript variants, *RP11-634B7.4-001* with 1541 bp and *RP11-634B7.4-002* with 722 bp^10^,^11^. Both have two exons and the resultant processed transcripts do not contain an open reading frame and are therefore not translated to proteins^10^,^11^. These long non-coding RNAs, however, are annotated as antisense to three olfactory receptor genes: *OR13G1*, *OR6F1* and *OR14A2*^10^,^11^. Antisense genes or transcripts are defined as those that overlap the genomic span that includes both exons and introns of a protein-coding locus on the opposite strand^11^. They also have antisense regulation of a coding gene, as reported in the literature^10^. In this case, the *RP11-634B7.4* gene, which contains the susceptibility loci for pre-treatment pain in HNSCC patients, can potentially regulate the *OR13G1*, *OR6F1* and *OR14A2* genes. A SNP, rs1151640, localized in *OR13G1*, results in a non-synonymous amino acid substitution (Ile132Val)^12^,^13^ and has been associated with chronic kidney disease^12^, coronary heart disease^14^ and myocardial infarction^13^. There are other olfactory receptor genes that have been implicated in various diseases such as fetal hemoglobin in sickle-cell anemia^15^, prostate cancer^16^ and pancreatic cancer^17^.

The olfactory receptor gene family is the largest gene family in the human genome^18^. It belongs to G-protein-coupled receptors^19^, a major class of sensory proteins that are therapeutic targets in pain pathways^20^. Although responsible for the recognition and G-protein-mediated transduction of odorant signals^21^, olfactory receptor genes may have dual olfactory and non-olfactory functions, or may have sole functions unrelated to olfaction^22^. They are specifically expressed in olfactory epithelium^19^, but their expression has also been verified in non-olfactory tissues like the tongue, heart, brain, spleen, pancreas, lung, kidney, breast, placenta, testis, spermatozoa, prostate, enterochromaffin cells, pulmonary neuroendocrine cells, and erythroid cells^22^,^23^.

Olfactory receptor genes have also been shown to affect pathways that involve mitogen-activated protein kinases (*MAPK/ERK*)^16^,^24^. For instance, it was demonstrated that odorant activated olfactory receptor genes activate MAP kinases^25^,^26^ that are important to olfactory sensory neurons, prostate cancer cell proliferation^16^, the wound healing process^27^, and hepatocellular carcinoma progression^28^. In our previous study, we identified a SNP within *MAPK1/ERK2* that was potentially associated with pre-treatment pain in head and neck cancer patients^29^ through a comprehensive literature search and gene network analysis (Ingenuity Pathway Analysis [IPA], Ingenuity^®^ Systems, www.ingenuity.com), as well as a genetic association analysis between the common SNPs within the IPA-derived genes and cancer pain in HNSCC patients. Because of the prior established effects of olfactory receptor genes on MAP kinases, we speculate that these olfactory receptor genes may be upstream regulators of *MAPK1/ERK2* in terms of eliciting pain signals associated with HNSCC.

Other genes that map to the region within 1M bases of the three SNPs, rs3862188, rs880143 and rs7526880, include *NLRP3, GCSAML* and *TRIM58. NLPR3* (The nucleotide oligomerization domain (NOD)-like receptor [NLR] family, pyrin domain containing 3), which is involved in the regulation of inflammation^30^, is an interesting molecule in pain studies. The *NLRP3* gene encodes a pyrin-like protein, a member of a protein family containing a pyrin domain, nucleotide-binding site domain and a leucine-rich repeat motif^30^, and has been associated with different inflammatory diseases^31^,^32^.

To our knowledge, this is the first report on the possible involvement of olfactory receptors in pre-treatment pain in HNSCC patients. There are limitations to our study. The level of cancer pain experienced prior to treatment varies among patients due to several factors such as the site and stage of disease. Our study did not have any restrictions on the site and stage of disease. Therefore, we further investigated the effect when adjusting for the TNM classification on the SNPs identified in the study using the 890 patients for which such data were available. Importantly, we observed similar association results whether or not we included the TNM classification as covariates. Therefore, we believe the SNPs identified in this study are associated with severe pain irrespective of the TNM classification. Also, our study population only involved self-reported Caucasians. GWASs that incorporate other races or ethnicities can further validate our results. Another limitation of this study is the relatively small sample size, which limited the statistical power for the analysis. We conducted a post hoc power analysis using the software program PS: Power and Sample Size Program^33^. Given 206 cases and 1,162 controls, we had 80% power to detect an OR of 2.58 for an association between a SNP and severe pre-treatment pain at the genome-wide significance level of 5 × 10^−8^ to account for multiple testing. Importantly, we employed a two-stage design, for which two independent data sets were used for the analysis, which helps control the false positive signals^34^,^35^. However, based on this relatively small sample size, we might have failed to identify additional SNPs that may be associated with pre-treatment pain in HNSCC patients. Nonetheless, we encourage future independent studies with larger sample sizes to further validate our results and possibly identify additional associated SNPs.

In conclusion, chromosome 1:44q may serve as a susceptibility locus for pain prior to treatment in HNSCC patients, with three variants, rs3862188, rs880143 and rs7526880, mapped to the *RP11-634B7.4* gene, a novel antisense gene to three olfactory receptor genes: *OR13G1*, *OR6F1* and *OR14A2*^10^,^11^. Consistent with previous studies, our current GWAS for pre-treatment pain in HNSCC patients supports the non-olfactory functions of olfactory receptor genes. Based on our findings from this study, together with the previously shown effects of odorant activated olfactory receptor genes on MAPK signaling pathways^16^,^24^,^25^,^28^, and our group’s recent report on *MAPK1/ERK2* as novel target genes for cancer pain^29^, it could be hypothesized that olfactory receptor genes, upstream effectors of the MAPK signaling cascade, may serve as novel target genes for pre-treatment pain in HNSCC patients. The transcriptional control mechanism of how the intronic variants rs3862188, rs880143 and rs7526880 affect pre-treatment pain in HNSCC patients is unknown. This observation is not unusual as most of the variants identified in the GWAS lie in the non-coding parts of the human genome, outside of the regions for which we know the function^36^, such as in the GWAS of pain severity in dysmenorrhea^37^ and of bipolar disorder^38^. The exploratory findings from this study require further experimental validation to explore biological mechanisms, which is key to defining the role of the intronic variants and non-coding RNA in HNSCC pre-treatment pain. Importantly, the evidence from experimental validations may provide insights into novel therapeutic targets for cancer pain management.

## Methods

### Study Population

The study population consisted of adult patients with newly diagnosed, histologically confirmed, previously untreated HNSCC. The patients were recruited for the study if they met the following criteria: a) newly diagnosed, untreated, histopathologically confirmed squamous cell carcinoma of the oral cavity, pharynx (excluding nasopharynx), or larynx; b) no previous cancers; c) age of 18 years or older; d) Texas resident; e) no blood transfusions in the past 6 months; and f) not on immunosuppression therapy. The patients were all previously untreated for cancer, which excluded pain associated with cancer treatment and focused on pre-treatment pain. This study was approved by the Institutional Review Board at MD Anderson Cancer Center and all procedures adhered to its guidelines and regulations, in accordance with the Declaration of Helsinki. All participants provided informed consent.

The collection of epidemiology and clinical data was conducted by staff interviewers when patients presented at MD Anderson, prior to being seen by clinicians. Specifically, pain “during the past week” was rated using a standardized 11-point numeric scale (0 = “no pain” and 10 = “pain as bad as you can imagine”), a recommended standard for pain assessment in clinical studies of pain^39^, at presentation of the patients before initiating cancer therapy. We considered a binary pain phenotype, where cases were individuals with severe pre-treatment pain (score ≥7) and controls were individuals with non-severe pre-treatment pain (score <7), based on the National Comprehensive Cancer Network cutoff score for severe pain^40^. From the patients whose pre-treatment pain information was available, we randomly selected individuals and assigned them to the discovery (~70%) or replication (~30%) phases of the GWAS, conditioning on identical percentages of males and females and similar distributions of age at diagnosis in the two phases^35^,^41^. Epidemiology and clinical data were also collected, including demographical factors such as age and sex as well as clinical factors such as TNM classification and comorbid conditions.

### Genotyping

Two hundred nanograms of DNA for each HNSCC patient were extracted from whole blood samples using the Qiagen QIAamp DNA Blood Maxi Kit following the manufacturer’s recommended protocol (Qiagen, Valencia, CA). High-throughput genotyping of the HNSCC patient samples for both phases was conducted at MD Anderson, using Illumina HumanOmniExpress-12v1 BeadChip (Illumina, San Diego, CA), according to Illumina protocols^42^. The HumanOmniExpress-12v1 BeadChip captures SNPs using a proven tagging SNP approach that has been successfully applied in hundreds of common variant GWAS^43^. We used Illumina’s BeadStudio for clustering and SNP calling^44^, based on the metrics listed in the SNP table of the BeadStudio software. All samples for each locus were used for clustering and thus the overall performance information was provided for each SNP locus. If no clusters were observed at a locus, we considered those SNPs as ‘no calls’, i.e., missing genotype. If the subset of loci were not clustered properly by the automated algorithm, the data were reviewed to identify loci that needed to be removed, manually edited or left unchanged. For all SNP assays, over 99% concordant results were obtained. Samples with overall genotyping rates <95% were excluded from the analysis.

### Quality Controls

We discarded SNPs based on the following criteria^41^: call rate <90%; minor allele frequency (MAF) <0.05; or *P* value of test for deviation from the Hardy-Weinberg proportion <1 × 10^−6^. Using the genotype data, we identified and excluded individuals with discordant sex information and duplicates from further analysis. To investigate the cryptic relatedness among individuals, we calculated the genome-wide identity-by-state distances on markers for each pair of individuals^45^,^46^. For any pair of individuals with allele sharing of >80%, we excluded the individual generating the lowest call rate. We also investigated the potential non-Western European ancestry for the individuals. In particular, we merged the cases and controls of our data with 2,502 reference samples from the 1000 Genomes Project data set (phase 3)^47^. For each pair of individuals, we calculated genome-wide identity-by-state distances on markers shared between the 1000 Genome samples and our SNP panel and used these as dissimilarity measures upon which to perform principal component analysis. All the quality control procedures described above were conducted using PLINK (v1.07)^48^.

### Statistical Analyses

Statistical analyses were conducted using PLINK (v1.07)^48^ and R software^49^. Deviation from the Hardy-Weinberg proportion for each SNP was assessed using the 1 degree-of-freedom *χ*^*2*^ test or Fisher’s exact test, where an expected cell count was <5^50^. The association between each SNP genotype and the pre-treatment pain status was assessed using multivariable unconditional logistic regression based on a 2-sided Wald test. Sex, age and information of clusters were included in the analyses as covariates. The information of cluster for each individual was obtained using a nearest neighbor cluster analysis based on genetic similarity. In particular, it was obtained using pairwise population concordance at *P* value < 0.005, with each cluster containing at least one case and one control. We performed a joint analysis that pooled data from both phases. To control for the possible confounding effects from two phases, in additional to sex, age and information of clusters, we included a fixed indicator to represent the phases as a covariate in the analysis and derived joint OR and 95% CI for each SNP and corresponding *P* value. We assumed an additive genetic model for each SNP when assessing associations. Special consideration was taken when analyzing the sex chromosomes. Only males were included for the association analyses between Y-chromosomal SNPs and the pre-treatment pain status. We conducted the statistical analysis in PLINK. For the association analyses between X-chromosomal SNPs and the pre-treatment pain status, we used the 1 degree-of-freedom *χ*^*2*^ test proposed by Clayton^51^, for which the males are treated as homozygous females. The analysis for the X-chromosomal SNP association included both males and females and was conducted using the R package “snpStats”^49^,^52^ that included Clayton’s test. We used the genome-wide significance *P* value threshold of 5 × 10^−8^ to account for multiple testing. Manhattan plots for the results from both phases and the joint analysis (Supplementary Fig. S1) were generated using R software^49^.

## Additional Information

**How to cite this article**: Reyes-Gibby, C. C. *et al.* Genome-wide association study suggests common variants within *RP11-634B7.4* gene influencing severe pre-treatment pain in head and neck cancer patients. *Sci. Rep.* **6**, 34206; doi: 10.1038/srep34206 (2016).

## Supplementary Material

Supplementary Information

## Figures and Tables

**Table 1:** Summary of results for SNPs associated with pre-treatment pain in HNSCC patients.

SNP	Chr.	Gene	Location (bp)*	MAF	Minor allele	Genetic model	Discovery phase		Replication phase		Combined	
							OR (95% CI)	*P*	OR (95% CI)	*P*	OR (95% CI)	*P*
rs3862188	1	RP11-634B7.4	247865773	0.28	G	ADD	1.73	7.35E-05	2.24	1.49E-03	1.87	3.45E-08
							(1.32–2.26)		(1.36–3.68)		(1.50–2.33)	
rs880143	1	RP11-634B7.4	247868113	0.28	G	ADD	1.73	7.35E-05	2.24	1.49E-03	1.87	3.45E-08
							(1.32–2.26)		(1.36–3.68)		(1.50–2.33)	
rs7526880	1	RP11-634B7.4	247862621	0.28	G	ADD	1.72	1.03E-04	2.24	1.49E-03	1.87	4.92E-08
							(1.31–2.26)		(1.36–3.68)		(1.49–2.33)	

^*^Human annotation release 105.
