# Supplementary material for: Genome-wide association study suggests common variants within RP11-634B7.4 gene influencing severe pre-treatment pain in head and neck cancer patients
Source: Sci Rep. 2016 Sep 27;6:34206. doi: 10.1038/srep34206 (PMC5037456; doi:10.1038/srep34206)

**Genome-wide association study suggests common variants within *RP11-634B7.4* gene influencing severe pre-treatment pain in head and neck cancer patients**

Cielito C. Reyes-Gibby, Jian Wang, Mary Rose T. Silvas, Robert K. Yu, Ehab Y. Hanna, Sanjay Shete

**Supplementary Table S1. Summary of results for 38 SNPs associated with pre-treatment pain in patients with squamous cell carcinoma of the head and neck patients with *P* values < 0.05 in the joint analysis that pooled data from both phases**


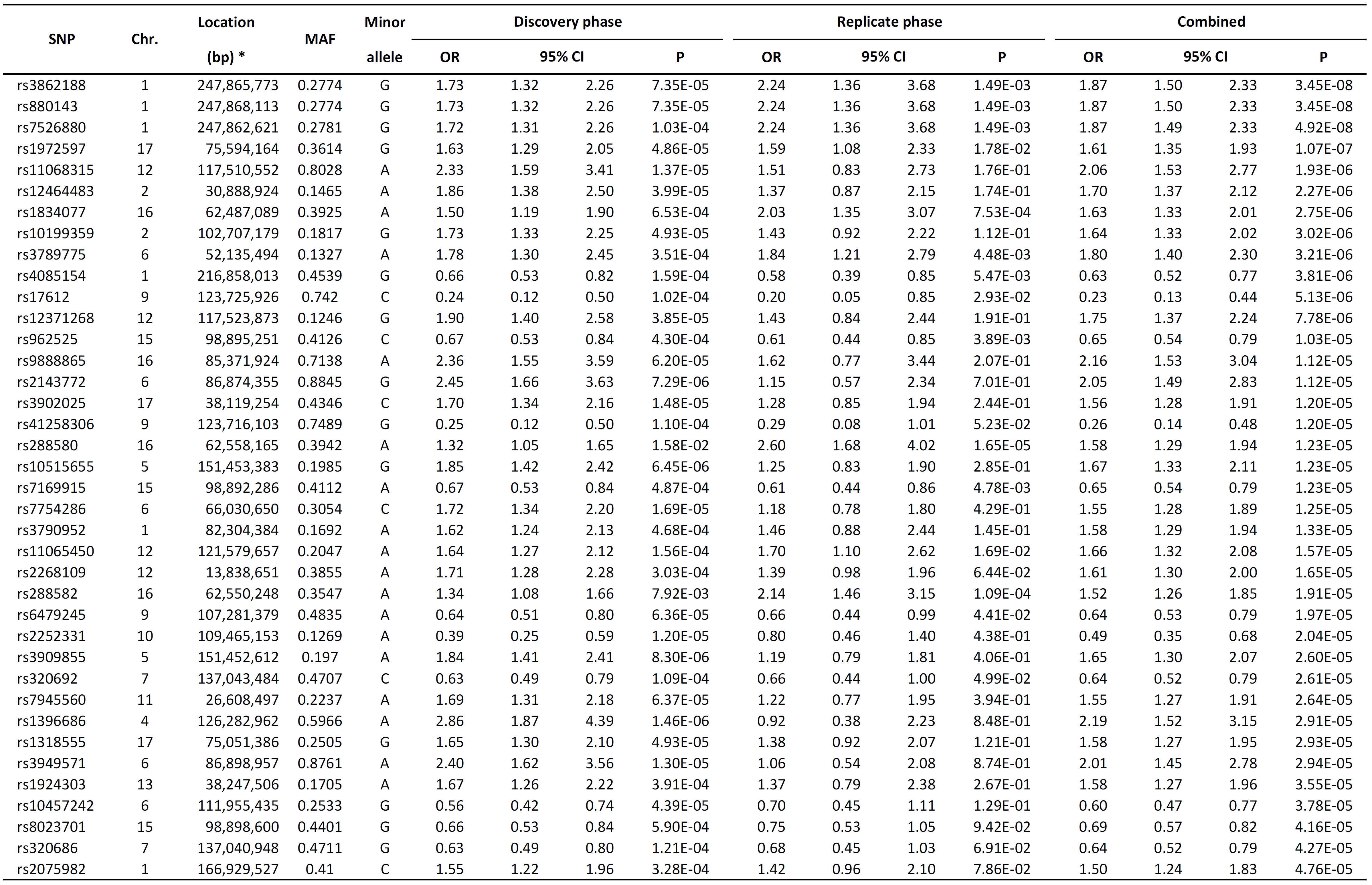
* Human annotation release 105

**Supplementary Figure S1. Manhattan plots of the GWAS of the pre-treatment pain in patients with squamous cell carcinoma of the head and neck, for discovery phase, replication phase and joint analysis, respectively**


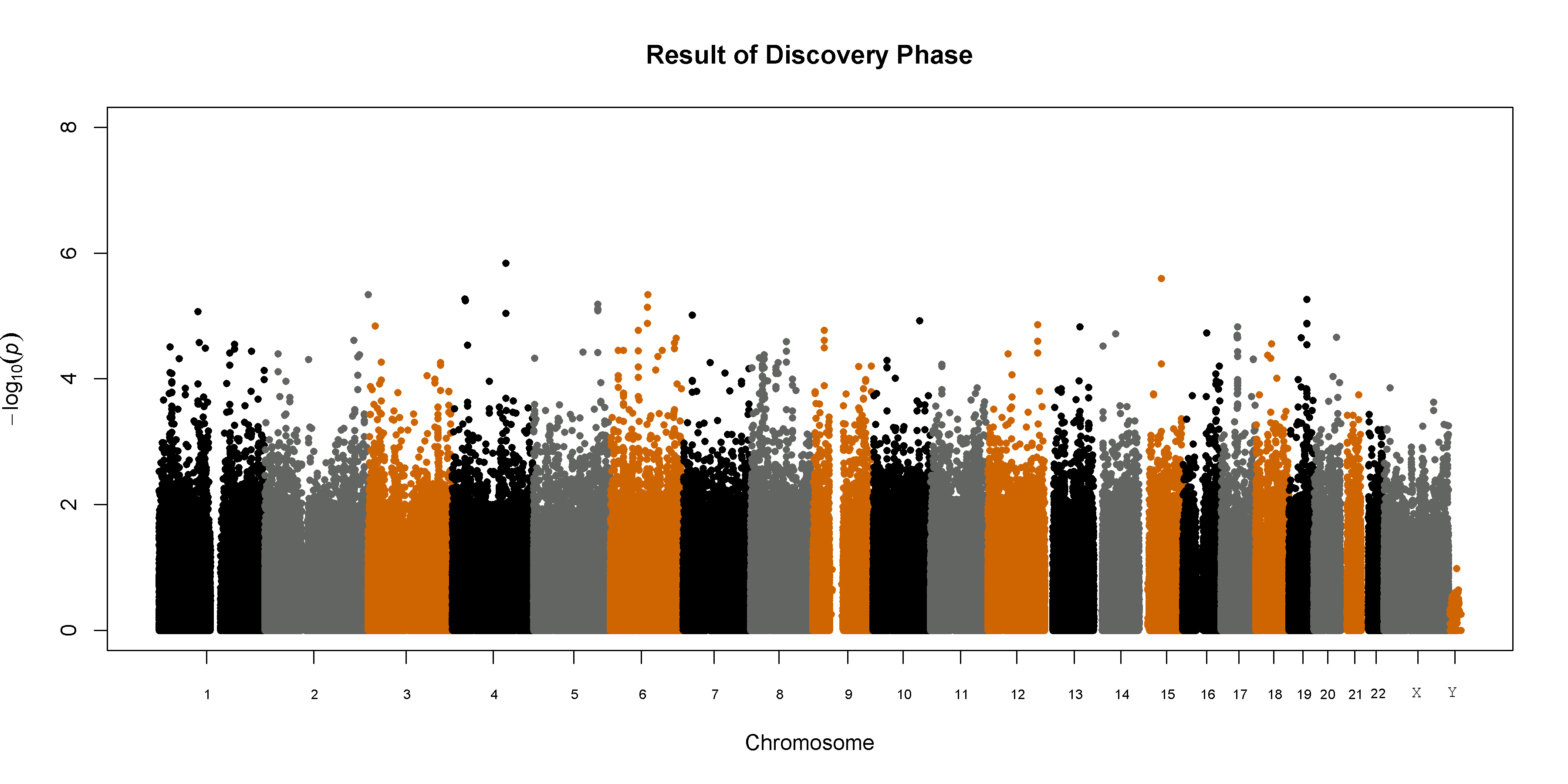


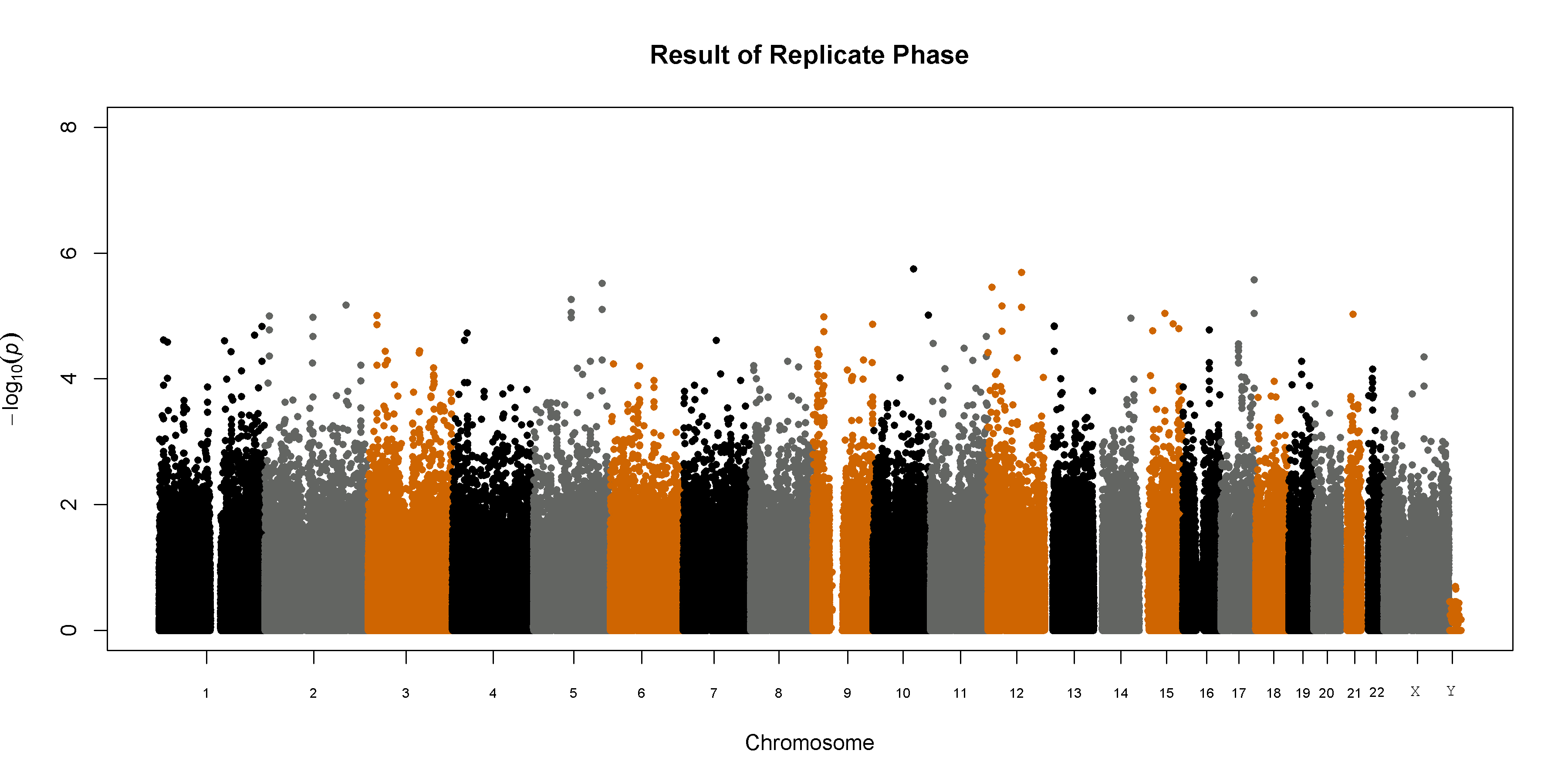


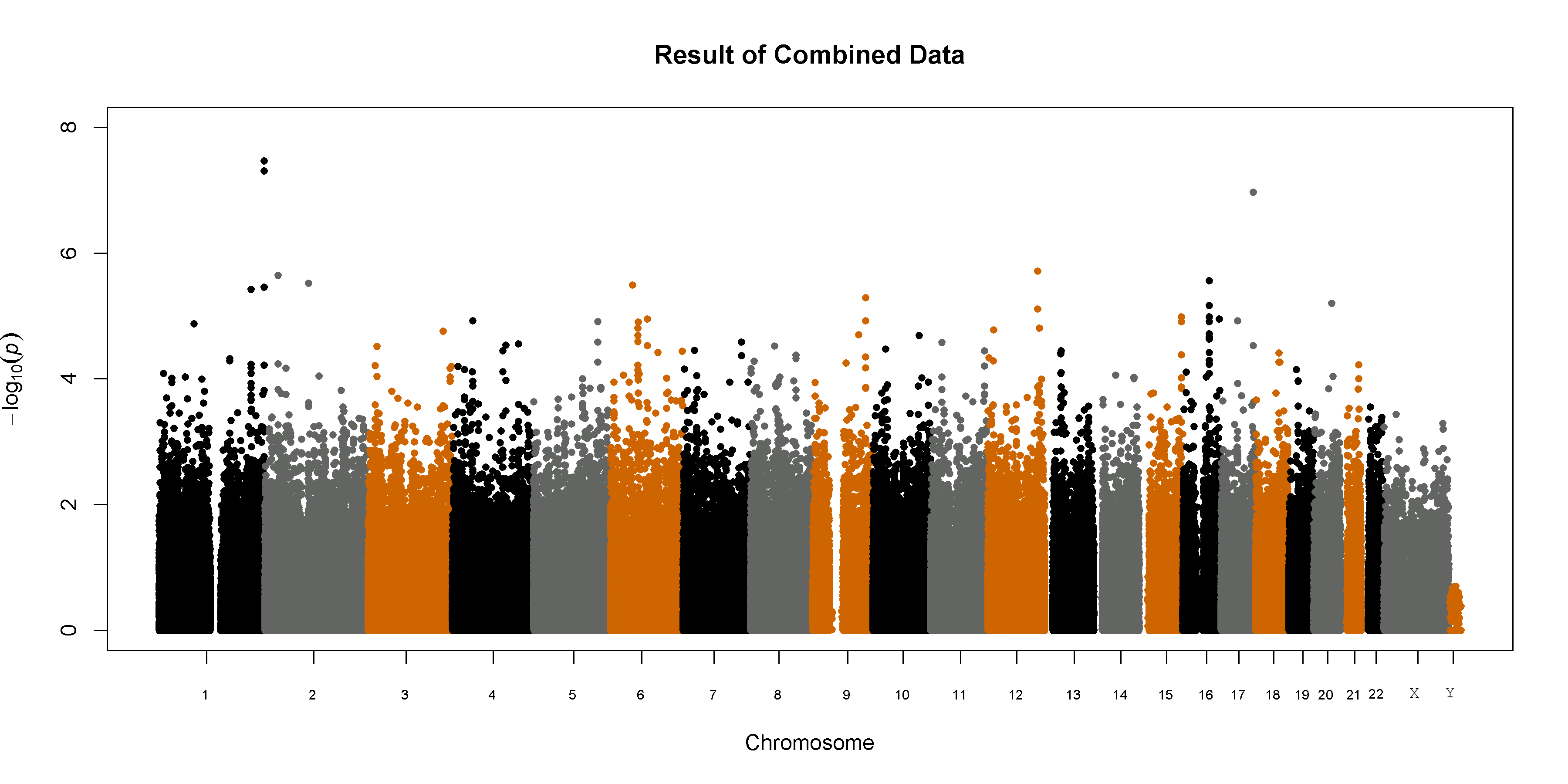

Supplement: Supplementary Information [file srep34206-s1.doc]
